# Consensus on a standardised treatment pathway algorithm for lumbar spinal stenosis: an international Delphi study

**DOI:** 10.1186/s12891-022-05485-5

**Published:** 2022-06-08

**Authors:** Christine Comer, Carlo Ammendolia, Michele C. Battié, André Bussières, Jeremy Fairbank, Andrew Haig, Markus Melloh, Anthony Redmond, Michael J. Schneider, Christopher J. Standaert, Christy Tomkins-Lane, Esther Williamson, Arnold YL. Wong

**Affiliations:** 1 Leeds Community Healthcare NHS Trust, Leeds, UK; 2 Faculty of Medicine, University of Leeds, Leeds, UK; 3 Faculty of Medicine, University of Toronto and Mount Sinai Hospital, Toronto, ON Canada; 4 Faculty of Health Sciences and Western’s Bone & Joint Institute, Western University, London, ON Canada; 5 School of Physical Medicine & Occupational Therapy, McGill University, Montreal, Canada; 6 Université du Québec À Trois-Rivières, Trois-Rivières, QC Canada; 7 Nuffield Department of Orthopaedics, Rheumatology and Musculoskeletal Sciences, University of Oxford, Oxford, UK; 8 Nuffield Orthopaedic Centre, Oxford Nuffield NHS Trust, Windmill Road, Oxford, UK; 9 Department of Physical Medicine and Rehabilitation, University of Michigan, Ann Arbor, USA; 10 Faculty of Health, Te Herenga Waka - Victoria University of Wellington, Wellington, New Zealand; 11 Institute of Health Sciences, Zurich University of Applied Sciences, Winterthur, Switzerland; 12 Curtin Medical School, Curtin University and UWA Medical School, University of Western Australia, Bentley, Australia; 13 Leeds Institute of Rheumatic and Musculoskeletal Medicine, University of Leeds, Leeds, UK; 14 Department of Physical Therapy, Clinical and Translational Science Institute, University of Pittsburgh, Pittsburgh, PA USA; 15 Department of Physical Medicine and Rehabilitation, University of Pittsburgh, Pittsburgh, PA USA; 16 Department of Health and Physical Education, Mount Royal University, Calgary, Canada; 17 Nuffield Department of Orthopaedics Rheumatology & Musculoskeletal Sciences, Centre for Rehabilitation Research, University of Oxford, Windmill Road, Oxford, UK; 18 Department of Rehabilitation Sciences, The Hong Kong Polytechnic University, Hung Hom, Hong Kong SAR, China

**Keywords:** Consensus, Delphi technique, Experts, Lumbar spinal stenosis, Algorithm, Clinical practice guideline

## Abstract

**Background:**

Lumbar spinal stenosis (LSS) is a common degenerative spinal condition in older adults associated with disability, diminished quality of life, and substantial healthcare costs. Individual symptoms and needs vary. With sparse and sometimes inconsistent evidence to guide clinical decision-making, variable clinical care may lead to unsatisfactory patient outcomes and inefficient use of healthcare resources.

**Methods:**

A three-phase modified Delphi study comprising four consensus rounds was conducted on behalf of the International Taskforce for the Diagnosis and Management of LSS to develop a treatment algorithm based on multi-professional international expert consensus. Participants with expertise in the assessment and management of people with LSS were invited using an international distribution process used for two previous Delphi studies led by the Taskforce. Separate treatment pathways for patients with different symptom types and severity were developed and incorporated into a proposed treatment algorithm through consensus rounds 1 to 3. Agreement with the proposed algorithm was evaluated in the final consensus round.

**Results:**

The final algorithm combines stratified and stepped approaches. When indicated, immediate investigation and surgery is advocated. Otherwise, a stepped approach is suggested when self-directed care is unsatisfactory. This starts with tailored rehabilitation, then more complex multidisciplinary care, investigations and surgery options if needed. Treatment options in each step depend on clinical phenotype and symptom severity. Treatment response guides pathway entrance and exit points. Of 397 study participants, 86% rated their agreement ≥ 4 for the proposed algorithm on a 0–6 scale, of which 22% completely agreed. Only 7% disagreed. Over 70% of participants felt that the algorithm would be useful for clinicians in public healthcare (both primary care and specialist settings) and in private healthcare settings, and that a simplified version would help patients in shared decision-making.

**Conclusions:**

International and multi-professional agreement was achieved for a proposed LSS treatment algorithm developed through expert consensus**.** The algorithm advocates different pathway options depending on clinical indications. It is not intended as a treatment protocol and will require evaluation against current care for clinical and cost-effectiveness. It may, however, serve as a clinical guide until evidence is sufficient to inform a fully stratified care model.

**Supplementary Information:**

The online version contains supplementary material available at 10.1186/s12891-022-05485-5.

## Background

Lumbar spinal stenosis (LSS) is a common cause of reduced mobility in older adults [1]. The prevalence and economic burden associated with LSS are expected to increase dramatically as our population ages [2–4]. Current treatment options are numerous and range from watchful waiting and a variety of non-surgical treatments to surgical interventions. There is a widely-recognised need for standardised and evidence-based clinical practice guidelines [5, 6] but current guidelines are inconsistent and rely on limited evidence [7]. The resulting unclear treatment pathways can lead to inefficient use of healthcare resources and ultimately impact negatively on patient care and outcomes.

Despite rising surgical rates in this patient group [2], up to 35% of patients may be less than satisfied with outcomes at one year following surgery [8]. Furthermore, early benefits of surgery over non-surgical treatment may fail to be sustained beyond 2–4 years [9, 10], and there is a 10% to 24% risk of complications from surgical intervention [11]. Although considered more urgent in cases of cauda equina symptoms or significant neurological deficits, surgery for LSS is generally regarded as an elective procedure for patients who have not achieved satisfactory outcomes with non-surgical care [2].

A variety of non-surgical treatments are frequently used to improve function and mobility, as well as to help manage LSS symptoms [12], but there is limited research evidence to support their use [13]. Treatment options include pharmacological interventions, education and lifestyle modifications, exercise programmes, manual therapy treatments, physical modalities, complementary therapies, walking aids/ orthoses, analgesic medications, medical interventional treatments such as spinal injections, psychologically-informed approaches and multimodal treatments comprising different combinations of these [13–15]. Whilst recent evidence supports some of these treatments [11], evidence remains patchy, and we still do not know which patients are likely to respond to which treatments [15], nor the most appropriate sequencing of these treatment options. Navigating this array of treatments is therefore challenging for both patients and clinicians.

In 2012, the *International Taskforce on the Diagnosis and Management of Lumbar Spinal Stenosis* was formed with the support of the *International Society for the Study of the Lumbar Spine* (ISSLS) to address some of the current challenges in LSS care. The Taskforce has already completed two phased Delphi studies achieving international expert consensus on diagnostic criteria for LSS [4, 16]. The current study follows on from these previous studies with the aim of reaching international expert consensus on an acceptable treatment algorithm to guide clinical practice. To our knowledge, this is the first study to apply the Delphi process in the development of a comprehensive treatment algorithm for patients with LSS.

## Methods

**Fig. 1:**
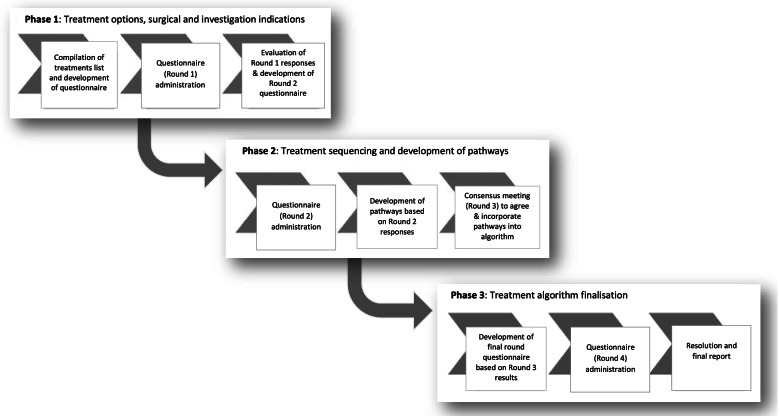
Diagrammatic overview of modified Delphi process

An outline of the three-phase modified Delphi method is presented in Fig. 1. An international study steering group provided oversight of the design, execution and data analysis according to proposed quality indicators for a Delphi study [17] on behalf of the *Taskforce*. The steering group comprised twelve clinical and research experts from surgical, physician and manual therapy/allied health professions across six countries (see Supplementary Table 1). In addition, patient and public representatives in the UK with LSS symptoms (CT, KR, RD, JuF) provided feedback at key stages of the study, optimising relevance of the algorithm to patient care and needs.

### Phase 1

The first phase focussed on establishing an agreed set of treatment options matched to descriptions of three LSS patient phenotypes. These phenotypes (A, B, and C described in Table 1) were developed by the study steering group based on common clinical symptoms in people with LSS.

**Table 1:** Lumbar spinal stenosis clinical phenotype descriptions

LSS Phenotype	Symptom Description
TYPE A: NEUROGENIC CLAUDICATION PAIN SYMPTOMS	*Neurogenic claudication* describes the typical widespread lower extremity pain aggravated during walking in people with LSS. Symptoms of aching, cramping, pain or burning most commonly affect both legs, though not always symmetrically. The symptoms are precipitated by standing as well as walking and relieved with sitting/ forward flexion/ lying down. These dynamic, posture-related bilateral pain symptoms are generally considered to be associated with central canal stenosis in the lumbar spine
TYPE B: NEUROGENIC CLAUDICANT SENSORY/ BALANCE SYMPTOMS	*Neuroischaemic* symptoms associated with LSS include tingling, paraesthesia, numbness and weakness in the lower extremities, usually bilaterally, and can also include problems with balance. As with other LSS phenotypes, these neuroischaemic symptoms are precipitated by standing and walking. The symptoms are generally considered to be due to central canal stenosis in the lumbar spine causing intermittent compromise of the cauda equina/ nerve roots related to compression, venous stasis and hypoxia
TYPE C: RADICULAR UNILATERAL LEG PAIN SYMPTOMS	*Radicular-type* leg pain symptoms affecting predominantly one lower extremity are generally considered to be due to direct nerve root compromise associated with lateral recess canal stenosis or foraminal stenosis in the lumbar spine. These symptoms generally follow a specific dermatomal pattern. Symptoms are aggravated by standing and walking but may also be present at other times. Since there may be inflammation of the nerve root, symptoms are less influenced by change in posture, and can also be experienced at rest, when sitting or at night in bed

In clinical practice, patients often present with a combination of these three overlapping symptom descriptions. The phenotype descriptions were developed as an aid to survey participants rather than a reflection of real patients who often present with a mixture of symptoms, and were expected to prompt different treatment selections. Bilateral neurogenic claudicant pain symptoms described in phenotype A might be associated with bilateral foraminal or lateral recess stenosis as well as with central canal stenosis

An initial consultation process used face-to-face and video-conference meetings within the study steering group and separately with two patient and public representatives (CT, KC) to compile a preliminary list of potential treatments. The list was then used to develop a questionnaire for the first online survey (Round 1) which sought to gain consensus on which of these potential treatments should be included for each phenotype.

We invited clinicians and researchers worldwide who identified themselves as having expertise in the management of LSS to take part in the survey. An invitation email providing an outline of the aim and content of the Delphi study and containing a link to the on-line survey questionnaire was distributed. The invitation was primarily through membership of relevant research and professional societies, such as the *International Society for the Study of the Lumbar Spine* (ISSLS), *International Forum on Back and Neck Pain Research in Primary Care*, and national societies contacted by members of the study steering group. In addition, musculoskeletal healthcare providers including physiotherapists, chiropractors, osteopaths, spinal surgeons, and physicians were invited through professional organisations. Experts in the field known to the study steering group were also invited through personal contact. To widen recruitment and reduce bias from the study steering group’s recruitment selection, we invited potential and recruited participants to share the email information and survey link with other suitable individuals known to them. The introductory page of the on-line questionnaire informed potential participants of the intention to publish non-identifiable data results from the study. Informed consent was required and gained online from all participants by completion of consent questions on the opening page prior to accessing the full survey questionnaire. The online surveys were developed and administered using Jisc Online Surveys tool (Jisc, One Castlepark, Tower Hill, Bristol, BS2 0JA, UK).

### Phase 2

The second phase of the study focussed on sequencing of treatments for the three different LSS patient phenotypes, refining the treatment pathways for each phenotype, and then incorporating the emerging pathways into an algorithm. This phase used both an online survey (Round 2) and an internal consensus process (Round 3).

The Round 2 survey questionnaire was designed by the steering group via an online meeting and subsequent in person meeting led by AW at the ISSLS meeting in Kyoto, and separate consultation with patient and public representatives (KC, CT). Invitations to participate in the Round 2 survey were distributed as in Round 1. Participants in this round were asked to rank all retained treatments from Round 1 for sequencing within a treatment pathway for each of the LSS phenotypes A, B and C. Response options ranged from ‘Always’ to ‘Never’ for various options relating to sequential steps within the pathway. Participants in both Rounds 1 and 2 were asked to consider available evidence, and to consider treatment accessibility, costs, and risks. Evidence was made available to participants through links embedded within the survey questionnaire. This evidence included high level systematic literature reviews produced by the Cochrane collaboration [11, 13, 18] and published clinical practice guidelines [19–21]. The aim was to facilitate the incorporation of current evidence into the development of treatment pathways, and to convey to participants that the process should be based on available evidence [22, 23].

A subsequent internal consensus round within the study steering group (Round 3) was held to refine the emerging pathways and ensure alignment with current evidence. This in-person consensus consultation was conducted through videoconference meetings and additional email correspondence to facilitate collective decisions when finalising a treatment pathway for each of the three LSS phenotypes A, B and C. Treatment options and sequencing in each pathway were agreed based on rankings from participants in Round 2, internal expertise, and available evidence. Clinical considerations (treatment costs, accessibility and risks) were again taken into account during this internal consultation round. The resulting treatment pathways for each phenotype were then combined into a single algorithm and, where clinically appropriate, treatment steps were aligned across the three phenotypes to reduce unnecessary complexity within the algorithm. Patient and public representatives (CT, JuF, RD) were consulted separately, and their suggestions were considered by the steering group and integrated into the proposed algorithm where appropriate.

### Phase 3

The third phase consisted of a final survey round (Round 4) to evaluate the level of consensus on the proposed algorithm and to elicit views about how useful the algorithm might be for different stakeholder groups.

After finalisation of the survey design by the steering group, invitations to participate in this final round survey were again distributed as in Rounds 1 and 2. For each of the external survey rounds (Rounds 1, 2 and 4) the questionnaires were open for up to 10 weeks (7 weeks for Round 1, 10 weeks for Rounds 2 and 3) to allow time for targeted societies and professional organisations to distribute the survey invitations to their membership.

### Data analysis

For each survey round, data from all completed questionnaires were analysed and used to generate a list of retained treatment items and pathway sequencing options for each LSS phenotype that were taken forward to the next round. Decisions were based on a predetermined consensus threshold of more than 70% [24] for retaining treatment/ sequencing options. If more than 70% of participants endorsed the treatment item/sequencing option and fewer than 30% rejected it, then the treatment item/sequencing option was retained. Conversely, if more than 70% rejected, or fewer than 30% endorsed a treatment/ sequencing option, then it was excluded. All other scoring patterns were taken to indicate non-consensus [25]. When there was no consensus or where evidence conflicted with consensus, decisions were made through internal agreement within the study steering group.

Descriptive statistics were used to analyse demographic data of participants and to analyse categorical variable responses, including frequency counts and percentage response rates against the defined consensus thresholds. Free text responses were analysed by coding responses into themes and subthemes using an inductive approach [26] by one author (CC) within an Excel spreadsheet to develop a summary of key themes. Coding and key themes were then sense checked by the study steering group.

## Results

### Phase 1

A list of 57 candidate treatments under 11 treatment category headings, and 8 investigation procedures were compiled from existing systematic review papers on LSS and from treatments suggested by the study steering group and patient and public representatives.

#### Round 1 survey

Among the 323 experts from 6 continents and representing 7 different professions who completed the Round 1 survey, consensus was achieved for inclusion of two treatment categories (advice and education; exercise) to be provided to all three LSS phenotypes; and for inclusion of multimodal care for phenotypes A and B; inclusion of manual therapy for phenotype C; and inclusion of investigations when clinically indicated across all three phenotypes. Two entire treatment categories (complementary therapies; physical modalities) received endorsement from fewer than 30% respondents for all three phenotypes leading to exclusion following this first round. A further 8 specific treatment types from several categories received < 30% endorsement for different phenotypes (Supplementary Table 2). All other treatment options were taken forward to Phase 2.

### Phase 2

#### Round 2 survey

Among the 159 survey respondents from 5 continents involving 7 different professional groups in this second round, it was agreed (based on > 70% consensus) that advice and education should always be provided for all three phenotypes. (Response options were ‘Always’; ‘As part of stepped care’; ‘As an optional adjunct’; ‘Only if new/worsening neurological deficit/cauda equina symptoms’; ‘Never’.) Consensus was achieved for manual therapy, medications, and spinal injections to be offered as part of stepped care for all three phenotypes and for walking aids to be part of stepped care for phenotype B based on < 30% endorsement of any other sequencing options (Supplementary Table 3). In addition, key indications for considering investigations and for spinal injection and spinal surgery were determined, each based on mean scores of > 7/10 on a 0–10 scale of agreement. For the remaining treatment options (exercise; psychologically informed care; walking aids for phenotypes A and C; and surgery), consensus was not achieved regarding when these treatments should be provided. Consensus was also not achieved on the sequencing of treatment options within stepped care. The most commonly endorsed sequencing selections were therefore taken forward to Round 3 (Supplementary Table 4).

#### Round 3 Internal Consensus

Decisions were made by the study steering group (Supplementary Table 5) to simplify the algorithm and to ensure available evidence was incorporated with the consensus results from Rounds 1 and 2. Feedback from patient and public representative included adding explicit wording that treatments should be tailored to individual needs, and adding information on falls within the ‘advice and education’ treatment category and was integrated into the proposed algorithm (Supplementary Fig. 1) for sharing with participants in the Round 4 survey.

### Phase 3

#### Round 4 survey

Final round surveys were completed by 397 clinical and research experts from 6 continents. Respondents represented 7 different professional groups, the majority (> 70%) with more than 15 years professional experience (Supplementary Table 1).

A total of 79% participants responded that they believe there is currently unwarranted variation in care for people with LSS, and 88% stated that they believe (40% definitely, 47% possibly/ to some extent) that the development and implementation of an internationally agreed treatment algorithm is likely to reduce this unwarranted variation in care. Survey responses are summarised in Table 2.

**Table 2:** Summary of responses Round 4

**Is there currently unwarranted variation in care for people with LSS?**	**Yes**		**No**
	**79**		**21**
**Is the development and implementation of an agreed algorithm likely to reduce unwarranted variation in care?**	**Yes definitely/ yes to some extent**	**Unsure**	**Definitely not/ probably not**
	**88**	**7**	**5**
**What is your overall level of agreement with the proposed treatment algorithm (scale 0–6)**	**Agree (score ≥ 4)**	**Neither agree nor disagree (score = 3)**	**Disagree (score ≤ 2)**
	**86**	**7**	**7**
**How useful do you think the proposed treatment algorithm will be for each stakeholder group? (0–6)**	**Agree (score ≥ 4)**	**Neither useful nor useless /unsure (score = 3)**	**Disagree (score ≤ 2)**
Private practice setting	**86**	7	7
Specialist/ secondary care setting	**83**	7	10
Primary care/ GP setting	**87**	8	5
Health service commissioners/providers	**74**	18	8
Healthcare policy makers	**74**	17	9
Healthcare researchers	**79**	15	6
Healthcare insurers	68	21	11
Patients (simplified version for SDM)	**83**	11	6

All values represent percentage of participants. Bold figures denote item responses reaching 70% consensus. *SDM *Shared decision making

Opinions about unwarranted variation in care differed between respondents, with not all professional or geographical subgroups reaching the 70% consensus threshold; only 66% respondents from Europe and 55% from Asia agreed that there is currently unwarranted variation in care, and only 54% physicians/physiatrists agreed. Similarly, only 54% surgeons and 61% physicians (physiatrists and family doctors/general practitioners) agreed that the development and implementation of a consensus treatment algorithm may reduce this unwarranted care, while 27% spinal surgeons and 46% physicians/physiatrists abstained from answering this question. Responses by profession and geographical location are summarised in Supplementary Table 6.

Overall opinion was in favour of the proposed algorithm with 86% of respondents rating their agreement with the algorithm 4 or above on a 0–6 scale. Of these, 22% rated their agreement as 6 (completely agree). A further 7% were unsure (neither agreed nor disagreed) and 7% did not agree with the algorithm (rated 0 to 2 on 0–6 scale) (see Table 2) Respondents from each of the six continents and from each professional group reached the threshold of 70% overall agreement for the algorithm (ratings of 4 or above on the 0–6 scale) (see Supplementary Table 6).

There was general consensus that the algorithm would be of use to healthcare clinicians working in each of various different clinical settings (83–87%), and that a simplified version would be useful as a shared-decision aid for patients/people with LSS symptoms (83%). There was also consensus that the algorithm would be of use to healthcare researchers (80%), and healthcare commissioners/providers (74%) but not to healthcare/ medical insurers. Key themes from comments for why respondents did not feel the algorithm was useful for specific stakeholders are summarised in Supplementary Table 8.

A table of respondents’ free text comments about the proposed algorithm organised under thematic headings is provided in Supplementary Table 7. The analysis of these comments identified three key themes:

i) Although there was overall consensus in support of the algorithm, there were mixed views expressed about how a standardised treatment algorithm fits in clinical practice alongside clinical reasoning based on experience and expertise, for example:

*‘An algorithm can’t replace clinical reasoning and clinical expertise’*

*(clinical physiotherapist, Europe, 21–25 years experience)*

*‘Treatment is mainly a question of experience’*

*(spinal neurosurgeon, public healthcare clinician, Europe > 25 years experience)*

*‘Not very useful in day-to-day practice. I think most practitioners are aware of these options and when in their clinical decision making to move the patient along.’*

*(clinical chiropractor in private practice, North America, > 25 years experience)*

*‘There will always be variations in preferred management methods based upon education and experience. Algorithms are valuable guidelines but still require individual interpretation’*

*(clinical chiropractor in primary/ community care, Austalia/NewZealand, > 25 years experience)*

*‘I worry about algorithms, but as a clinical reasoning framework I think this could be very helpful especially to support those who may not see cases frequently and this puts LSS on the map’*

*(physiotherapy researcher, Europe, > 25 years experience)*

*‘Ultimately, a consensus treatment pathway will provide the healthcare services and their patients with improved understanding, greater standardisation of care and provide a model for best practice. Everyone involved in spinal stenosis will benefit.’ (clinical physiotherapist in private practice, Europe, >25years experience)*

ii) Views relating to specific treatments and treatment sequencing were identified among different professions, for example comments from surgeons about non-surgical treatment steps in the pathway before surgical treatment/ opinion included:

*‘As an orthopaedic spinal surgeon, who treats LSS regularly…. if implemented at the primary care level, will help reduce the amount of refers for patients with manageable symptoms’*

*(orthopaedic spinal surgeon clinician and researcher, North America, 0–5 years experience)*

*‘I would be afraid that many patients would never see a surgeon’*

*(spinal neurosurgeon, clinician, Europe, 16–20 year experience)*

*‘The main point is that the algorithm suggests surgery in rare cases only and after numerous, mostly useless, conservative interventions’*

*(orthopaedic spinal surgeon, clinician and researcher, Europe, 16–20 years experience)*

iii) Implementation issues were identified, with comments particularly focusing on resources and on validation and evidence, for example:

*‘The algorithm needs to be validated with the treatment effects of the patients’ (orthopaedic spinal surgeon, clinician and researcher, Europe, > 25 years experience)*

*‘This is an excellent pathway if all elements of services are available in a timely fashion’*

*(clinical physiotherapist in primary/ community care, England, > 25 years experience)*

*‘Requires equity in international resource distribution/ availability, i.e. infrastructure, workforce, expertise etc. This is different in different countries with different health systems, cultures, beliefs and economies. Might be applicable in developed countries to a certain extent…. Will be a struggle to fulfil parts of the algorithm in countries with struggling economies…’*

*(clinical physiotherapist, primary/ community care, Europe, 21–25 years experience)*

*‘Many of the proposed steps are not accessible in this order in the UK National Health Service (NHS) due to local commissioning pathways already in place, making implementation difficult’*

*(clinical physiotherapist, public healthcare, Europe, 6–10 years experience)*

**Fig. 2:**
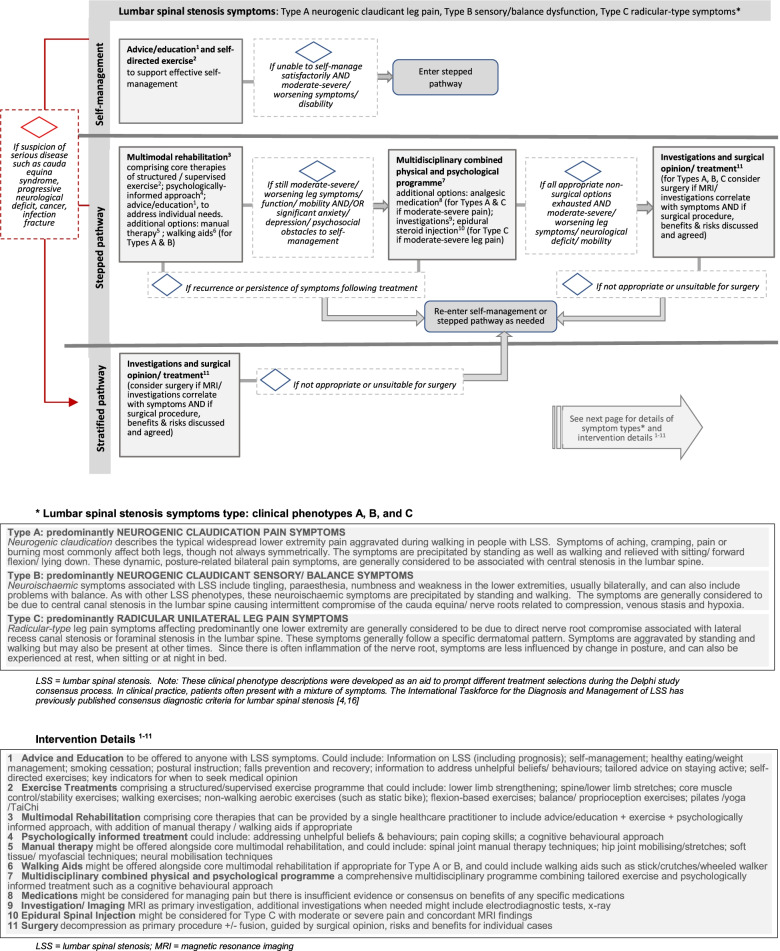
Proposed treatment algorithm. Page 1: treatment pathway options for people with lumbar spinal stenosis symptoms Page 2: symptom phenotypes and intervention details

Other comments from participants referred to the LSS phenotype descriptions; suggestions for additional information in the algorithm relating to assessment, diagnosis, treatments and timelines. The final version of the proposed algorithm (Fig. 2) addresses comments on the layout and presentation of the algorithm.

## Discussion

This study integrates worldwide clinical and research opinion, the views of patient and public representatives, and evidence-based oversight from a dedicated international study steering group to develop a consensus treatment algorithm for people with LSS symptoms. The proposed algorithm presented in Fig. 2 aims to help guide clinical care and combines both stratified and stepped care approaches. It advocates immediate imaging/ surgical opinion for those with specific clinical indications, whilst for others a stepped care approach or self-directed care is suggested, taking into consideration different LSS phenotypes. For those following the stepped pathway, tailored multimodal rehabilitation involving exercise and education is advocated in step one, with more complex multidisciplinary care (step 2) or imaging / investigations and surgery (step 3) being considered if required. The pathway is nonlinear and treatment options can be followed in a step-wise fashion or entered at any point depending on clinical need and response to previous treatment, and on suspicion of serious disease.

Stratified and stepped care are different approaches that can help direct more intensive treatments to patients who need them and will benefit from them whilst avoiding overtreatment or exposure to risk for those who require less intensive interventions or no treatment [27]. Stratified care assumes that patients who will benefit from more intensive or invasive treatments without the risk of harm or overtreatment can be identified early. Stepped care, on the other hand, assumes a substantial number of patients will benefit from less intensive and invasive treatments and will not be harmed by a delay in accessing more intensive treatments. In the absence of sufficient research evidence to inform fully stratified patient-centred pathways [5, 6, 15], combining stepped and stratified approaches and integrating support for self-management may be the most promising option for improving current delivery of care with the minimum burden or risk to patients [28].

The nature and severity of LSS symptoms and disability varies widely between individuals, presenting different treatment needs that can change over time. Our study considers three common LSS patient phenotypes and explores a wide range of treatment options that might target individual needs at different times. With limited research evidence to inform clinical practice guidelines, even the most recently published guidelines offer sparse detail to guide treatment across the LSS disease spectrum [19, 21]. Moreover, an evaluation of guidelines published to date highlights inconsistent recommendations which are potentially biased towards surgical treatments [7]. In clinical practice, people with LSS are seen across all tiers of the healthcare system and might be directed to diverse treatments depending on the experience and expertise of different healthcare practitioners rather than patient needs. It is unsurprising, then, that our study captured a widespread perception that there is unwarranted variation in current care, and that this might be improved by the development and implementation of an agreed treatment algorithm.

Previously published algorithms lack detail across the range of LSS symptoms and treatment options and/ or have been developed without consensus [29–32]. The consensus process is underpinned by the assumption that the opinions of many outweigh those of the individual [33]. In the absence of consistent level-1 evidence in the research literature, our modified Delphi approach leveraged the combined expertise and opinions of international spine researchers and clinicians, providing validity for the proposed algorithm as a framework to guide clinical care [34]. We used anonymous online surveys distributed through multi-professional research societies and professional organisations. This allowed us to access widespread opinion that was not influenced by the views of other respondents. By focusing first on which treatment options to include, and secondly on treatment sequencing for each LSS phenotype in Rounds 1 and 2, we were able to aggregate a large volume of relevant expert knowledge. Importantly, patient and public representative involvement was embedded at each stage of the study [35] with the aim of optimising relevance and acceptability of the algorithm for people with LSS. In addition, whilst acknowledging that the current evidence is limited, survey participants were encouraged to refer to available evidence when responding to questions because this has been shown to influence the decisions made [36] and to result in higher quality tools for guiding clinicians [37]. Furthermore, we used the expertise of the study steering group to ensure up-to-date evidence-based decisions at each stage of the study. Through online and in-person meetings, the steering group was able to explore opinions, modify responses, and achieve internal consensus to finalise the proposed algorithm during Round 3 [38] before wider-level evaluation of agreement with the algorithm in the final Round 4.

Whilst the proposed algorithm largely aligns with and expands upon recommended treatments in current guidelines, some decisions in the treatment pathways warrant further discussion. Despite a paucity of evidence in LSS research, participants in our study reached 90% agreement that appropriate advice/education and exercises should always be made available to people with LSS presenting with all three phenotypes and all levels of symptom severity. Advice/education is considered an important aspect of supporting self-management for chronic musculoskeletal conditions in general [39] and is included in recommendations from the current Danish and Canadian guidelines for LSS [19, 21]. However, there is no agreed source of clear and comprehensive advice and education for patients with LSS and such resources may yet need to be developed using appropriate coproduction methods. Exercise treatments, often incorporated in multimodal non-surgical treatment packages, are recommended in some clinical guidelines [19, 21]. Emerging evidence suggests supervised exercise with manual therapy may be more effective than self-directed care [40] but remains insufficient to inform the most effective treatment combination or to determine the optimum exercises type or dosage. Despite the limited evidence, our results suggest a widespread view that exercise is a key component of multimodal non-surgical treatments for people with all types of LSS symptoms.

Both complementary therapies and physical modality treatments were excluded from treatment pathways for all three phenotypes in Round 1 of the survey due to endorsement from fewer than 30% participants. There was considerable discussion and debate within the study steering group and contradictory comments from survey participants regarding several of the remaining non-surgical treatments retained from Rounds 1 and 2. These included psychologically informed approaches, manual therapy, medications and spinal injections. Limited evidence and sometimes conflicting guidance [19, 21, 41] mean that the value of these treatments remains contentious in the management of LSS. For example, a recent review highlights recommendations both for and against manual therapy in different clinical practice guidelines [7]. Based on survey responses endorsing manual therapy in some phenotype presentations and recent evidence-based guidance [21], it was decided to include manual therapy as an optional part of multimodal care in our proposed algorithm. Medications were also included because survey participants did not exclude this treatment category at any point in the consensus process. However, the study steering group decided that there was inadequate evidence [7, 21, 41] or participant agreement to advocate any specific medications in the proposed algorithm. Whilst psychologically informed treatments are not found in LSS clinical practice guidelines, two recent trials include a psychologically informed approach as part of multimodal care for people with LSS [42, 43]. Based on internal consensus, limited evidence, and survey participant endorsement of between 45 and 61% for different phenotypes presentations, the study steering group agreed to include psychologically-informed approaches in Step One and a multidisciplinary combined physical and psychologically-informed programme in Step Two of the proposed algorithm. This is in agreement with UK-based recommendations in the national back and radicular pain pathway based on National Institute for Health and Care Excellence (NICE) guidance [44]. Spinal injections were excluded from the algorithm for neurogenic claudication phenotypes based on recent evidence-based guidance [21] and internal consensus, but were included as an option for patients with radicular-pain type symptoms, again mirroring UK recommendations in the national back and radicular pain pathway [45]. Comments from participants and lack of consensus on selection/ sequencing of these treatments underlines the differing views and need for further research evidence to guide the use of these non-surgical treatment options in clinical practice.

For surgical treatment, the most popular sequencing options selected by study participants were either as part of stepped care, or in the presence of specific clinical indications (‘severe/ worsening neurological deficit’ and ‘cauda equina syndrome’ reached mean scores of 77% and 93% respectively). This suggests clear agreement on the stratified pathway in our proposed algorithm, which directs patients with these ‘red flag’ indicators straight to imaging and surgical opinion. However, consensus did not reach the 70% threshold regarding the sequencing position of surgery within the stepped care pathway for the majority of patients without these surgical indicators for any of the three phenotypes. The varied and sometimes strong views expressed by participants in our study reflects the ongoing debate around the emphasis on surgical treatments compared to non-surgical options in clinical guidelines [7].

Despite variation in opinion on specific treatments and use of algorithms in clinical practice, there was overall consensus in support of the proposed algorithm. Respondents from Asia were least likely to agree with the algorithm or to believe it would reduce unwarranted variation in care. This may reflect geographical differences in clinical practice and in clinical phenotype presentations; the proportion of patients presenting with cauda equina type symptoms due to genetic influences on spinal canal area may present specific challenges in Asian populations. Furthermore, concerns were expressed suggesting that availability of resources, reluctance of certain professions, and access to specific treatments might present challenges for implementation in different countries and healthcare settings.

Several limitations about this study need to be acknowledged and discussed. First, our inclusive definition of ‘experts’ when recruiting survey participants may be viewed by some as insufficiently stringent, but reflects our desire to capture a broad range of relevant opinions. Response numbers from different professions and geographical locations were also imbalanced despite our best efforts to target survey distribution, potentially leading to some bias in our results. Second, the length of the second round survey questionnaire that focussed on treatment sequencing may have deterred participants from completing this round. This would explain the lower number of responses in this survey round. Third, the use of an algorithm to represent expert treatment opinions may be seen as too simplistic or too rigid for adoption in clinical practice. On the other hand, it offers an easily followed visual guide that busy clinicians who need rapid access to guidance might find useful. Comments from participants suggest that a simple visual reference aid like this is valued by some and might be particularly helpful in non-specialist primary care settings. Finally, whilst the proposed algorithm may help to guide clinical decision-making with patients, it has not been validated in a clinical setting and may not be feasible to adopt or appropriate for widespread implementation in its current form. Further research is needed to evaluate clinical and cost effectiveness of the treatment pathways for different clinical phenotypes in the proposed algorithm compared to current care. Despite the above noted limitations, the key strengths of this study include the contribution of several hundred multi-professional spine experts around the world to the development of an agreed treatment algorithm to guide clinical care for people with different LSS symptoms.

## Conclusion

Our results highlight wide recognition that there is currently unwarranted variation in care for people with LSS which may be improved by the development of an internationally agreed treatment algorithm to guide clinical care. The proposed treatment algorithm developed in this study through international expert consensus combines a stratified approach when first-line imaging and surgical consideration are indicated, and a stepped treatment approach for others when supported self-management is not sufficient. Availability of advice, education and exercise are advocated for all to aid self-management. The stepped approach starts with individually tailored rehabilitation, and also offers multidisciplinary care combining physical and psychological approaches, and finally surgery if indicated. The pathway suggests stepping up and down indications with entrance and exit points based on response to previous treatment, symptom severity, functional limitations, and suspicion of serious disease. The proposed algorithm was widely perceived by study participants to be of use for clinicians in a range of settings and as a tool to aid shared decision-making with patients.

Although the proposed algorithm is not intended as a treatment protocol and is yet to be evaluated against current care for clinical and cost effectiveness, in the absence of level-1 research evidence for most treatments, it may be a helpful guide to assist in clinical decision-making until more robust evidence is available to inform a fully stratified care model. In the long-term, standardised treatment options targeting individual needs based on an agreed symptom-based treatment algorithm could lead to more efficient use of healthcare resources and better patient outcomes.

## Supplementary Information

**Additional file 1: Supplementary Table 1.** Details of study participants. **Supplementary Table 2.** Summary of consensus achieved Round 1 - Intervention options for three LSS phenotypes. **Supplementary Table 3.** Summary of consensus achieved Round 2 - Intervention sequencing. **Supplementary Table 4.** Summary of sequencing selections based on consensus or most common responses. **Supplementary Table 5.** Internal consensus decisions for final proposed algorithm. **Supplementary Table 6.** Opinions about algorithm by profession and by geographical region. **Supplementary Table 7.** Themes identified in respondent comments. **Supplementary Table 8.** Themes from respondents’ comments on reasons why algorithm not useful each stakeholder group. **Supplementary Figure 1.** Draft algorithm shared in Round 4.

## Data Availability

The datasets generated and/or analysed during the current study are available in the Leeds Research Data Repository (RADAR), DOI https://doi.org/10.5518/1106
