# Supplementary material for: Consensus on a standardised treatment pathway algorithm for lumbar spinal stenosis: an international Delphi study
Source: BMC Musculoskelet Disord. 2022 Jun 8;23:550. doi: 10.1186/s12891-022-05485-5 (PMC9175311; doi:10.1186/s12891-022-05485-5)
Supplement: Supplementary file 1 — Additional file 1: Supplementary Table 1. Details of study participants. Supplementary Table 2. Summary of consensus achieved Round 1 - Intervention options for three LSS phenotypes. Supplementary Table 3. Summary of consensus achieved Round 2 - Intervention sequencing. Supplementary Table 4. Summary of sequencing selections based on consensus or most common responses. Supplementary Table 5. Internal consensus decisions for final proposed algorithm. Supplementary Table 6. Opinions about algorithm by profession and by geographical region. Supplementary Table 7. Themes identified in respondent comments. Supplementary Table 8. Themes from respondents’ comments on reasons why algorithm not useful each stakeholder group. Supplementary Figure 1. Draft algorithm shared in Round 4. [file 12891_2022_5485_MOESM1_ESM.docx]

**Supplementary Table 1: Details of study participants**

| **External surveys rounds** | **Round 1**  Treatment options | **Round 2**  Treatment sequencing | **Round 4**  Overall agreement |
| --- | --- | --- | --- |
| **Survey dates**  Opening date  Closing date  **Total number or respondents**  **Professional background**  Chiropractors  Physiotherapists  Osteopaths  Spinal orthopaedic surgeons  Spinal neurosurgeons  Physiatrist/Physician/  Family doctors/general practitioners  Other  **Clinical or Research expertise**  Clinician  Researcher  Clinician and Researcher  Other  **Geographical location**  North America  South America  Europe  Asia  Australasia  Africa  Other  **Primary work setting**  Public healthcare  Private healthcare  Academic institute  Other  **Experience**  Qualified 15 years +  Regularly assess/ manage people with LSS  **Conflict of interest declared** | 07-08-2019  18-10-2019  100 **(323)**  35 (113)  44 (142)  0.5 (1)  14 (45)  1 (5)  4 (13)  0.5 (1)  1 (3)  65 (209)  3 (11)  29 (93)  3 (10)  64 (206)  1 (1)  21 (69)  9 (31)  3 (11)  2 (5)  27 (86)  47 (153)  23 (73)  3 (11)  68 (221)  77 (250)  2 (2) | 18-01-2020  31-03-2020  100 (**159)**  33 (53)  40 (64)  1 (1)  14 (23)  4 (6)  6 (10)  1 (1)  1 (1)  63 (100)  7 (11)  30 (47)  39 (62)  0 (0)  28 (45)  12 (20)  20 (31)   1. (1)   0 (0)  28 (45)  47 (74)  23 (37)  2 (3)  73 (116)  73 (116)  2 (1) | 23-04-2021  30-06-2021  100 **(397)**  24 (97)  31 (123)  2 (9)  21 (83)  17 (65)  3 (13)  1 (3)  1 (4)  75 (296)  2 (10)  30 (91)  22 (88)  1 (2)  59 (235)  7 (29)  8 (31)  2 (8)  1 (4)  44 (176)  38 (150)  17 (69)  1 (2)  71 (282)  79 (312)  0.3 (1) |
|  | *Percentage of respondents (number), except for survey dates* | | |
| **Study steering group for preparatory work Phase 1, Round 3 internal consensus, and study oversight** | | | |
| CMC: Physiotherapist, Clinician and researcher, Europe (UK), 10 LSS publications  CA: Chiropractor, Clinician and researcher, North America (Canada), 23 LSS publications  MB: Physiotherapist, Researcher, North America (Canada); 15 LSS publications  AB: Chiropractor, Researcher, North America (Canada), 3 LSS publications  JF: Surgeon (retired), Clinician and researcher, Europe (UK), 7 LSS publications  AH: Physiatrist/Physician, Clinician and Researcher, North America (United States), 39 LSS publications  MM: Surgeon and rheumatologist, Clinician and Researcher, Australasia (New Zealand), 22 LSS publications  MJS: Chiropractor, Researcher, North America (United States), 13 LSS publications  CJS: Physician, Clinician and Researcher, North America (United States), 7 LSS publications  CT: Kinesiologist, Researcher, North America (Canada), 35 LSS publications  EW: Physiotherapist, Researcher, Europe (UK), 4 LSS publications  AYLW: Physiotherapist, Researcher, Asia (Hong Kong), 2 LSS publications  JuF: patient/public representative, Oxford, UK  RD: patient/public representative, Oxford, UK  CT: patient/public representative, Leeds, UK  KC: patient/public representative, Leeds, UK | | | |

*Note: Affiliations for the steering group members are detailed in the author list on title page*

*LSS = lumbar spinal stenosis*

**Supplementary Table 2: Summary of consensus achieved Round 1 - Intervention options for three LSS phenotypes**

| **Intervention** | **Consensus level** | **Comments** |
| --- | --- | --- |
| **Interventions to be included** | | |
| Diagnostic Investigations: **MRI scan** | 94% | 78% endorsed use of MRI investigation when specific clinical indications are present |
| **Advice and Education** for phenotypes A, B, C | 87-93% across phenotypes | consensus not achieved on individual components of advice/ education |
| **Exercise treatments** for phenotypes A, B, C | 89-92% across phenotypes | consensus not achieved on individual exercise elements |
| **Multimodal treatment** for phenotypes A and C | 85% | multimodal treatment to include advice/ education, exercise, and manual therapy |
| **Manual therapy** for phenotype C | 72% | consensus not achieved on individual manual therapy techniques |
| **Intervention categories or specific interventions to be excluded** | | |
| **Complementary therapy**: whole treatment category excluded for phenotypes A, B, C | 60% rejected and  <30% endorsed across all phenotypes |  |
| **Physical modalities**: whole treatment category excluded for phenotypes A, B, C | 52% rejected |  |
| From manual therapy category: **lumbar spinal traction** excluded for phenotypes A, B | <30% endorsed for phenotypes A, B | consensus not achieved on other individual manual therapy treatments |
| From aids/orthotics category: **lumbar corset/brace** excluded for phenotypes A, B, C | <30% endorsed for phenotypes A, B, C | consensus not achieved on other aids/ orthotics other than walking aids (see below) |
| From aids/Orthotics category: **walking aids** excluded for phenotype C | <30% endorsed for phenotype C | consensus not achieved on other aids/ orthotics) |
| From psychologically informed treatments category: **neuroscience education; motivational interviewing; acceptance commitment therapy** excluded for phenotypes A, B, C | <30% endorsed for phenotypes A, B, C | consensus not achieved for other psychologically informed treatments such as cognitive behavioural therapy |
| From investigations category: **CT; US; myelogram; ABPI; Doppler** excluded | <30% endorsed |  |
| From surgical interventions category: **spinal cord/dorsal column stimulator; interspinous spacer implant** excluded | <30% endorsed |  |
| *Consensus based on 70% or more endorsement for interventions to be included, and based on criteria of <30% endorsement and/or >45% rejection for interventions to be excluded;* | | |

*LSS= lumbar spinal stenosis; MRI = magnetic resonance imaging; CT = computerized tomography; US = ultrasound; ABPI = ankle/brachial pulse index*

**Supplementary Table 3: Summary of consensus achieved Round 2 - Intervention sequencing**

| **Intervention sequencing/ indications** | **Consensus level** |
| --- | --- |
| **Advice and Education: should always be offered for phenotypes A, B, C** | 90-93% endorsement across phenotypes |
| **Key indications for diagnostic investigations** cauda equina syndrome signs/symptoms; to exclude serious pathology/for differential diagnosis; to plan surgery/injection; if significant/ worsening neurological deficit; severe symptoms and disability; unsatisfactory outcome from conservative care | Mean score of >7/10 agreement on 0-10 scale |
| **Key indications for spinal surgery**: cauda equina syndrome, motor deficit, imaging matching symptoms, low surgical risk, unsatisfactory outcome from conservative care | Mean score of >7/10 agreement on scale of 0-10 |
| **Key indications for spinal injection**: severe radicular symptoms, unsatisfactory from non-invasive care | Mean score of >7/10 agreement on scale of 0-10 |
| Exercise excluded from ‘never’, ‘only if new/worsening neurological deficit’, ‘cauda equina symptoms’ or ‘optional adjunct’ options in pathway, and might therefore be offered ‘always’ OR as ‘part of stepped care pathway’ | <30% for excluded sequencing options |
| Psychologically informed treatments excluded from ‘never’, ‘only if new/worsening neurological deficit’, cauda equina symptoms, or ‘optional adjunct’ options, and might therefore be offered ‘always’ OR as ‘part of stepped care pathway’ | <30% for excluded sequencing options |
| **Manual therapy** excluded from ‘always’, ‘never’, ‘only if new/worsening neurological deficit’, ‘cauda equina symptoms’ or ‘optional adjunct’ options in pathway, and might therefore be offered as ‘**part of stepped care pathway’** | <30% for excluded sequencing options |
| **Medications** excluded from ‘always’, ‘never’, ‘only if new/worsening neurological deficit’, ‘cauda equina symptoms’ or ‘optional adjunct’ options in pathway, and might therefore be offered as ‘**part of stepped care pathway’** | <30% for excluded sequencing options |
| **Walking aids** excluded from ‘always’, ‘never’, ‘only if new/worsening neurological deficit’, ‘cauda equina symptoms’ options in pathway for phenotype A and might therefore be offered as an ‘an optional treatment’ or as ‘part of stepped care pathway’ for phenotype A. Excluded additionally from ‘optional treatment’ for phenotype B, and might therefore be offered as ‘**part of stepped care pathway’ for phenotype B** | <30% for excluded sequencing options |
| **Spinal injection** for phenotype C excluded from ‘always’, ‘never’, ‘only if new/worsening neurological deficit’, ‘cauda equina symptoms’ or ‘optional adjunct’ options in pathway, and might be offered as ‘**part of stepped care pathway’** | <30% for excluded sequencing options |
| Surgery excluded from ‘always’, ‘never’ or ‘optional adjunct’ options in pathway, and might therefore be offered as ‘part of stepped care pathway’ OR ‘if indicated due to severe/worsening neurological deficit/Cauda Equina syndrome’ | <30% for excluded sequencing options |
| *Consensus based on 70% or more endorsement, or based on ruling out sequencing endorsed by <30% respondents* | |

**Supplementary Table 4: Summary of sequencing selections based on consensus or most common responses**

| **Most commonly endorsed sequencing selections** |
| --- |
| Exercise treatments: Always – for phenotypes A,B,C, mild-moderate and moderate-severe symptoms* |
| Multimodal care of Advice/education + exercise + psychologically informed treatment: Step 1 for types B and C, and for type A with moderate-severe symptoms. |
| Multimodal care of Advice/Education + exercise: Step 1 for type A with mild-moderate symptoms |
| Manual therapy: Step 1 in stepped care pathway for phenotypes A,B,C any severity |
| Psychologically informed treatments: Step 1 in stepped care pathway for phenotypes A,B,C any severity |
| Walking aids: Step 1 or Step 2 in stepped care pathway for phenotypes A or B |
| Medications: Step 2 in stepped care pathway for phenotypes A,B,C any severity |
| Spinal injection: Step 2 or 3 for phenotype C depending on symptom severity |
| Surgery: Final step in stepped care pathway OR when indicated by severe/ worsening neurological deficit/ Cauda Equina syndrome |

*Note: *Sequencing selection based on consensus. All other selections based on most common responses*

**Supplementary Table 5: Internal consensus decisions for final proposed algorithm**

| Finalised decisions for treatment algorithm following internal consensus study steering group meetings | |
| --- | --- |
| **Red Flag Pathway** | • red flag pathway to be followed if there is suspicion of serious disease, such as cauda equine syndrome, progressive neurological dysfunction, cancer, infection, fracture |
| **Advice and education:** | . to be offered to all people with LSS symptoms prior to entering stepped care pathway  • to include: Information on LSS, self-management, healthy eating/weight management,  smoking cessation, postural instruction, falls prevention and recovery (as suggested by patient and public representatives), information to address unhelpful beliefs/ behaviours; tailored advice on staying active; self - directed exercises; in addition, the steering group agreed that the information should include key indicators for when to seek medical opinion  • to exclude ‘advice to rest’ and ‘avoidance of symptom provocation’. It was agreed by the steering group to include ‘tailored advice on staying active’. |
| **Escalation** into stepped care pathway | should be considered if  • moderate-severe/ worsening symptoms/ disability AND  • unable to self-manage satisfactorily following advice/education |
| **Stepped Care pathway: Step 1** | **Multimodal care**  • to be defined as a package of core therapies provided by a single healthcare practitioner  • to include i) Advice/Education; ii) Structured/supervised exercises; iii) Psychologically informed care (such as a cognitive behavioural approach)  • may also include manual therapy as option for all phenotypes, but only if combined with advice/education, exercise, and psychologically informed care (see below)  • may also include walking aids for Type A or B (see below)  **Exercise Treatments**  • to comprise a structured/supervised exercise programme that could include: lower limb strengthening; spine/lower limb stretches; core muscle control/stability; walking exercises; non-walking aerobic exercises (such as static bike); flexion-based exercises; balance/ proprioception exercises; pilates/yoga/TaiChi  **Psychologically informed treatment**  • could include: a Cognitive Behavioural approach; addressing unhelpful beliefs & behaviours; pain coping skills  **Manual therapy**  • could include: spinal joint manual therapy techniques, hip joint mobilising/stretches, soft tissue/ myofascial techniques, neural mobilisation techniques, but NOT traction  **Walking Aids**  **.** could include walking aids such as stick/crutches/wheeled walker for Type A |
| **Escalation** into Step 2 of stepped care pathway | If after Step One care, still presenting with  • moderate-severe/worsening leg symptoms/ function/mobility AND/OR  • significant symptoms of anxiety/depression, psychosocial obstacles to self - management AND  • unable to self-manage satisfactorily |
| **Stepped Care Pathway: Step 2** | **Combined physical and psychological programme**  • to be defined as a comprehensive multidisciplinary programme combining tailored exercise, physical treatments and a cognitive behavioural approach preferably in a group context  **Medications**  • insufficient evidence or consensus on benefits of any specific medications  **Spinal Injections**  • epidural spinal injection might be considered for Type B if moderate-severe pain AND  concordant MRI findings |
| **Escalation** into Step 3 of stepped care pathway | If all appropriate Step 1 and Step 2 options exhausted AND  • moderate-severe/worsening leg symptoms/neurological deficit/ mobility AND  • unable to self-manage satisfactorily |
| **Stepped Care Pathway: Step 3** | **Surgery**  • surgical treatments might be considered for all phenotypes if concordant imaging AND surgical procedure, benefits & risks discussed & agreed  • could include: decompression +/- fusion guided by surgical opinion for individual cases  **Investigation/ Imaging**  • to be considered if suspicion of serious disease, AND/OR  • planning injection or surgery.  • MRI scan, +/- additional investigations such as x-ray, electro-diagnostic tests if indicated |
| **Additional points** | • treatment pathway to be circular rather than linear to allow provide flexibility in options  • re-entry to self-management or stepped care pathway can be considered at any point  • reference for red flag indicators to be included in algorithm and references (Finucane, L., et al. "International Framework for Red Flags for Potential Serious Spinal Pathologies." *Journal of Orthopaedic & Sports Physical Therapy* 0 (2020): 1-23) |

**Supplementary Table 6: Opinions about algorithm by profession and by geographical region**

| **Responses by profession** | | | | | | | | |
| --- | --- | --- | --- | --- | --- | --- | --- | --- |
|  | **Physiatrist/ physician**  **(n = 13, 3%)** | **Chiropractor**  **(n = 97, 24%)** | **Spinal orthopaedic surgeon**  **(n=83, 21%)** | **Spinal neurosurgeon**  **(n = 65, 17%)** | **Physiotherapist/ physical therapist**  **(n = 123, 31%)** | **Osteopath**  **(n= 9, 2%)** | **GP/family doctor**  **(n= 3, 1%)** | **Other**  **(n= 4, 1%)** |
| **Is there currently unwarranted variation in care? (yes)** | 54* | 81 | 70 | 75 | 82 | 89 | 100 | 100 |
| **Is algorithm likely to reduce unwarranted variation? (yes to some extent/ definitely)** | 54* | 73 | 59* | 49* | 80 | 78 | 67* | 75 |
| **Overall agreement with algorithm (score ≥4)** | 92 | 89 | 82 | 75 | 93 | 78 | 100 | 50* |
| **The algorithm is likely to be useful for** | | | | | | | | |
| **Private practice setting** | 77 | 91 | 86 | 75 | 89 | 89 | 100 | 75 |
| **Specialist/ secondary care setting** | 77 | 86 | 82 | 65 | 89 | 100 | 100 | 75 |
| **Primary care/ GP setting** | 85 | 89 | 82 | 77 | 94 | 100 | 100 | 75 |
| **Health service commissioners/providers** | 77 | 77 | 70 | 61* | 84 | 56* | 66* | 25* |
| **Healthcare policy makers** | 69* | 78 | 72 | 54* | 86 | 56* | 67* | 75 |
| **Healthcare researchers** | 85 | 88 | 75 | 71 | 86 | 67* | 100 | 75 |
| **Healthcare insurers** | 77 | 77 | 65* | 54* | 71 | 89 | 33* | 75 |
| **Patients (simplified version for SDM)** | 85 | 83 | 79 | 65* | 94 | 89 | 100 | 75 |
|  |  |  |  |  |  |  |  |  |
| **Responses by geographical location (continent)** | | | | | | | |  |
|  | **Europe**  **(n = 235, 59%)** | **N.America**  **(n = 88, 22%)** | **S.America**  **(n = 2, 1%)** | **Australia/NZ**  **(n = 31, 8%)** | **Asia  (n = 29, 7%)** | **Africa**  **(n = 8, 2%)** | **Other**  **(n = 4, 1%)** |  |
| **Is there currently unwarranted variation in care? (yes)** | 77 | 81 | 100 | 77 | 76 | 75 | 75 |  |
| **Is algorithm likely to reduce unwarranted variation? (yes to some extent/definitely)** | 66* | 75 | 100 | 71 | 55* | 75 | 75 |  |
| **Overall agreement with algorithm (score ≥4)** | 85 | 97 | 100 | 77 | 72 | 88 | 75 |  |
| **The algorithm is likely to be useful for:** | | | | | | | |  |
| **Private practice setting** | 84 | 95 | 100 | 81 | 72 | 100 | 100 |  |
| **Specialist/ secondary care setting** | 80 | 91 | 100 | 81 | 72 | 100 | 100 |  |
| **Primary care/ GP setting** | 86 | 95 | 100 | 84 | 72 | 75 | 100 |  |
| **Health service commissioners/providers** | 72 | 84 | 100 | 77 | 59* | 50 | 75 |  |
| **Healthcare policy makers** | 72 | 82 | 50* | 81 | 72 | 75 | 100 |  |
| **Healthcare researchers** | 75 | 93 | 100 | 81 | 79 | 88 | 75 |  |
| **Healthcare insurers** | 63* | 83 | 100 | 71 | 62* | 88 | 50* |  |
| **Patients (simplified version for SDM)** | 81 | 89 | 100 | 84 | 76 | 88 | 100 |  |

*All values represent percentage of respondents. * values not reaching 70% consensus level; GP= general practice; SDM = shared decision-making; NZ = New Zealand*

**Supplementary Table 7: Themes identified in respondent comments**

| **Theme 1: How a standardised treatment algorithm fits in clinical practice** | | | | | | | | | | | |
| --- | --- | --- | --- | --- | --- | --- | --- | --- | --- | --- | --- |
| **Algorithm concept overall: need/usefulness** | | | | | **Standardised care vs non-standardised/individual care** | | | **Utility for different stakeholders** | | | |
| This just describes the sensible treatment options for patients. | | | | | I think there is a danger of over simplification as each patient presents with different severity symptoms and signs. | | | I think it is more likely to benefit GP's and non-specialist services in decision making/referral route. Specialist centres know the route/options for these common presentations. | | | |
| I think it is very helpful in indicating what comprehensive treatment should look like | | | | | algorithms only help so far personalized medicine approaches are required | | | I struggled with the secondary care one. Surely if the algorithm is being followed they would only be seeing the final 2 steps + serious? I guess it would be useful for them to make sure patients have had the right treatment before they have surgery/injections? | | | |
| The algorithm applies in its context. Some of the patients we receive have no understanding of condition, the GPs and patients expectations are for a fix of the problem. The presenting age group has multi-morbidity. After having factored everything in and the psychosocial barriers to accepting the care we are left with an individualistic care package. | | | | | The treatment is mainly a question of experience. There are many cases which surprises between the picture of the spine and the development of the disease. | | | the algorithm is useful for patients and maybe useful for general practitioners | | | |
| Ultimately, a consensus treatment pathway will provide the healthcare services and their patients with improved understanding, greater standardisation of care and provide a model for best practice. Everyone involved in spinal stenosis will benefit. | | | | | Still it's very individualised treatment depending on radio. Findings, severity of symptoms, comorbidities and social parameters | | | Other stakeholder groups may have an interest in the results but it is always the clinician together with the patient to decide. | | | |
| I really like the staged approach with clear multimodal management but built in safety to ensure escalation/change in pathway as needed…. reduced clinical variations and provided clear guidance of what management should be. | | | | | An algorithm can't replace clinical reasoning and clinical experience. | | | In Germany the specialised spine clinics are the last in the cascade of treatment. They are not meant to provide care of step 0-2a. | | | |
| I just think any system to aid best management and audit and research is great | | | | | In my daily practice with LSS patients I see an urgent need to develop an unité de doctrine amongst everybody involved with care for these patients | | | To be frank: If a specialist really needs that algorithm to avoid a wrong or inefficient treatment of his patients, he should wonder to quit his job. | | | |
| Believe it to be useful, yet other factor may play a critical role | | | | | algorithms only help so far personalized medicine approaches are required | | | It has to accommodate for a large cohort of professionals and therefore varied opinions. | | | |
| too complex and prescriptive for my practice | | | | | not really helpful, clinical individual decision | | | Professional clinicians usually understand enough that the guidelines have little purpose whereas the policy and decision makers could probably benefit from guidance | | | |
| Excellent conceptually with clear pathway. | | | | | Guidelines are good, but human beings are individuals and have the right to search for different treatment options | | | I feel a simpler version would be good for many osteopaths and their patients to work with together. | | | |
| Deep down, I worry about algorithms, but as a clinical reasoning framework I think this could be very helpful especially to support those who may not see cases frequently and this puts LSS on the map | | | | | there will be patients with varying degrees of symptom severity and pattern which necessitate the use of clinical judgement in spite of the algorithm | | | If you are specialised on spine surgery, this algorithm gives no help, because it is too unspecific. Treatment options are clear anyway. | | | |
| The principle is clear and well known to the specialists. For them it is not specific/individual enough. Because what is best in average might not be the best for the individual case. Therefore this Algorithm may only be a fair direction of the course. | | | | | Some cases are very individual and difficult to represent in an algorithm | | | Health insurers need guidance to form policy as this is a complex issue. | | | |
| There will always be variations on preferred management methods based upon education and experience. Algorithms are valuable guidelines but still require individual interpretation. | | | | | This flowchart is a good start, but often times these are used more to limit care or coverage. Patient conditions and situations are extremely variable and can’t be easily placed in this. | | | Researchers will gain the most use (benefit) from this flowchart. | | | |
| Reduction of the cost. Better chance for target patients. Efficient management of cases in need. | | | | |  | | | I don't see insurers or policy makers changing their current payment policies to reflect what is best for the patient. | | | |
| some algorithm is required | | | | |  | | | The lesser the experience the more useful the information | | | |
| The algorithm will not be able to show me the right decision. Maybe it is something for artificial intelligence. | | | | |  | | | Not sure health care insurers should be involved with spinal pathways. Private practitioners will find this either very beneficial as it will give them and their clientele a clear understanding of their own responsibility to their condition, or find it loses them cause for further interventions. OPD Physios, Spinal specialists in primary care and interface settings will find this very useful as a tool to inform and manage the huge number of referrals from GP services that do not understand the condition. GPs are often too busy to read the policies. | | | |
| There should be an evidence based pathway to standardise treatment that is clinically and cost effective | | | | |  | | |  | | | |
| it is very useful to know when to treat and when to refer and to whom | | | | |  | | |  | | | |
| A thorough evidence-based infographic similar to this utilized by all stakeholders would be phenomenally helpful. | | | | |  | | |  | | | |
| This represents a best practice algorithm per my experience in clinical practice | | | | |  | | |  | | | |
| Should be considered beginner level information well below the understanding of specialist level providers i.e. the more exposure you have to these patients the greater the awareness of these basic concepts. | | | | |  | | |  | | | |
| I’ve only see flowcharts such as these successfully used in EMS for rapid triage or emergent situations. Not very useful in day-to-day practice. I think most practitioners are aware of these options and when in their clinical decision making to move the patient along. | | | | |  | | |  | | | |
| This is the pathway for care and recovery for individuals with LSS, which would be useful for all healthcare practitioners to be on the same pathway. | | | | |  | | |  | | | |
| The present algorithm contains a good full understanding of pain and spinal causes most associated to cause them. | | | | |  | | |  | | | |
| Not generally useful, does not direct treatment and is overly complicated | | | | |  | | |  | | | |
| this is the problem with algorithms. Although often misinterpreted, clinicians have a tendency to start at the beginning and rigidly follow it through step by step while the patient suffers unnecessarily while being denied the treatment most likely to help them. | | | | |  | | |  | | | |
| Guidance regarding treatment options is always helpful in planning care options and discussing evidence based care with patients. | | | | |  | | |  | | | |
| **Theme 2: Specific treatments and treatment sequencing** | | | | | | | | | | | |
| **Stepped approach and stratified approach** | **Self-management and multimodal rehabilitation/ core therapies** | | **Spinal Injections** | | | **Surgical treatment** | **Combined psychological and physical programme and medical care** | | **Imaging** | | **Manual therapy** |
| I don't think it should be a stepped pathway. Surgery should be an option at any stage of the pathway as long as it is backed up by severe symptoms, concordant imaging and full discussion over the options/risks/benefits etc. | I am concerned about the overlap of stage one and two | | I agree with the pathway as shown but was surprised by the lack of injection therapy option for those with claudicant stenosis given many older patients with stenosis and significant comorbidities may not be surgical candidates? | | | i am more convinced of operation results | I feel that CPPP is not appropriate for patients with spinal stenosis, if they are a suitable surgical candidate with correlating imaging. | | ...imaging too late | | I do not like the inclusion of the term 'manual therapy' as it denotes hands on care which is not necessary (or helpful) for some of the items included within this. I suggest renaming as 'adjunctive therapy'. |
| I think that if patients have features of neurogenic claudication of any sort and they don’t settle with combined conservative management then they should (if they wish) see a surgeon. The stepped care model presented seems too long to me. | Not convinced about the wait and see self-management approach (particularly for type C). I would prefer an intervention period (even if very brief) followed by self-management | | Steps 1 and 2 should be amalgamated. epidural injections don't work, transforaminal injections for radiculopathy have some effect | | | I would be afraid that many patients would never see a surgeon. | Impractical where I practice (Greater Toronto Area) because psychological therapy is scarcely available. | | The cause of the MRI-stenosis is the key for decision-making. MRI-findings without proper x-Ray are worthless, | | I would like to see manual assistance used earlier as a preventative rather than symptomatic approach |
| I wonder if some patients with severe symptoms would necessarily go through each of the steps, rather jump straight to imaging and possible surgical opinion (following shared decision making conversation)? | Conservative management if diagnosed early with patient education would probably have a superior outcome to early surgical or invasive treatment. | | I disagree with the use of injections for stenosis symptoms in the absence of radicular symptoms, and in patients with multi-level stenosis | | | too conservative approach | In my experience I don't think and don't utilise CPPP type interventions for LDSS-I don't think I have encountered much psychological distress in this group. Maybe because I am in 2nry care and only see those who would benefit from surgery | | Should recommend scan early - 6 weeks if not resolving | | I'm not sure about the use of manual therapy but the rest seems good. |
| ... the completion of all steps could probably oppose a pathway too long for severe pain and inability to walk. | Step 1 is no long-term definitive treatment option. | | NICE NG59 does not recommend epidural for LSS, as a provider of spinal injections I know that they offer short term relief. | | | Surgical treatment should be offered at an earlier stage of treatment because of a low risk profile and a high satisfaction rate. | psychological methods won't be helpful at all for Schizas C and D stenosis | |  | | I would place more emphasis on the provision of manual therapy in particular approaches that offer a combination of flexion, distraction and mobilisation combined with exercise. |
| treatment depends on severity of symptoms correlating with severity of stenosis in MRI, | To have therapy options without regard of the cause of the disease won't work sustainably. | | I would only recommend one try of conservative treatment with ESI for progredient type b. If this one try fails, I would recommend the operation to prevent SCI | | | There are several options to treat: endoscopic, microscopic decompression, spacer, interlaminar Fusion, 360° decompression+ fusion and dynamic stabilization+ decompression. The decision making has to be done individually, an algorithm would not respect it and give you an artificial feeling of safety. | Content fine except not detailing medicine recommendations. Analgesic medicines are started but not further described e.g. absent in intervention details section. | |  | | ...manipulative therapy (Chiropractic) can be started and should be encouraged during self-management stage. |
| As a thought, might want to try and stratify care for those likely to need more earlier on | The non-operative stage should be single, using all modalities available, and then, escalate to surgical options if failure to respond. | | I think that injections techniques like epidural injections and facet joint injections are far more helpful than indicated in this algorithm and are also useful in Type A and B, depending on MRI findings. | | | The main point is that the algorithm suggest surgery in rare cases only and after numerous, mostly useless, conservative interventions. | For my opinion you have to combine Step 1 with Analgesic medication, otherwise it'll be not suitable for the patients | |  | | It disregards the details of manual therapy which is able to provide control for the majority of early to moderate cases. |
| The red flag pathway I agree with | I would welcome adjuncts such as weight loss programmes, smoking cessation etc. | | I don´t understand that only in group C peridural injections should be considered | | | Surgery should not be offered as the last treatment option |  | |  | |  |
| I agree with the interventions as described but do not agree that a patient has to have exhausted steps 1 and 2 in order to be considered for surgery. If symptoms and disability are severe enough with concordant imaging, surgery should be offered | Why treat so conservatively with a one way disease. Intervene early and aggressively. | | epidural steroid injection also works for Type A | | | Elements of spinal decompression provide an excellent outcome if patients are able to implement homecare concurrently. |  | |  | |  |
|  | The goal should be ... ability to manage conservatively for as long as possible which takes patients being accountable and diligent with their self-directed exercises. | | I generally think of ESI as even less effective for central. | | | The algorithm spends a great deal of time on general health advice before reaching the step that is necessary for significant symptoms - surgery! |  | |  | |  |
|  | Step 2 does not make too much sense to me. The stenosis is a mechanical problem, mostly caused by bony or cartilaginous hypertrophy, therefor rather irreversible. An overly extended conservative therapy is only indicated in cases of comorbidities with high perioperative risks. | |  | | | As an orthopaedic spinal surgeon who treats LSS regularly, this is a very logical and easy to follow pathway that, if implemented at the primary care level, will help reduce the amount of refers for patients with manageable symptoms. |  | |  | |  |
|  | In my opinion a therapeutic trial of those conservative modalities should preclude the offering of spinal surgery for the majority of patients. | |  | | |  |  | |  | |  |
|  | I am not convinced that the elderly patients will be able to do so much physiotherapy and or multimodal treatment. | |  | | |  |  | |  | |  |
|  | i believe that the self-management pathway should be altered. | |  | | |  |  | |  | |  |
|  | The decision maker should be the patient, many of whom are incensed by the death spiral of physiotherapy they get steered towards. | |  | | |  |  | |  | |  |
|  | There are three steps to non-operative treatment. No indication of patient education. It’s a bit like an arthritic hip. If you educate patients They will know when they want surgery. | |  | | |  |  | |  | |  |
|  | Sometimes the patients also only wants to know, why they cannot walk. | |  | | |  |  | |  | |  |
| **Theme 3: Implementation issues** | | | |  | | | | |  | | |
| **Implementation in general** | | | | **Implementation: evidence/ validation** | | | | | **Implementation: resources** | | |
| a simplified decision tree on GP primary acre IT system | | | | further research is needed in this area to validate efficacy | | | | | I imagine in the real world of delivery (NHS as I am UK based) this will create long delays and add to covert rationing. | | |
| The algorithm has to be self-explanatory and easy to understand. The decision categories be simple to use. | | | | Very difficult to give definite conclusions as many mechanisms determining severity and progress are ill understood so premature adoption of an algorithm could diminish exploration at individual or service level. As an example, the limitation of epidural injection to Type C patients may be informed by inaccurate concepts of causation and scanty outcome data. If this prematurely dubbed some treatment as non-compliant with accepted guidelines, then it would not be useful. | | | | | Noble idea but requires equity in international resource distribution/availability, i.e. infrastructure, workforce, expertise etc. This is different in different countries with different health systems, cultures, beliefs and economies. Might be applicable in developed countries to a certain extent (also limitation in resources) and will be a struggle to fulfil parts of the algorithm in countries with struggling economies hence the health service. That said, ambition and innovation towards better health is commendable. | | |
| i think spinal services may well have established practices in place (good/ bad) but may be more resistant to adopting an external policy. | | | | The algorithm suggests procedures that are commonly used but lack evidence and are even off label use (epidural corticoid injections). | | | | | Impractical where I practice (Greater Toronto Area) because psychological therapy is scarcely available. | | |
| I feel more familiarity is needed with algorithm | | | |  | | | | | In my area we were providing CBT cut due to funding 3+ years ago, we were doing epidurals but suspended 18 months ago, Community back pain clinic finally funding suspended April this year | | |
| I need to test it out | | | |  | | | | | Main problem is that there is no availability of the resources required and therefore, in practice, the guidelines are not as useful as they should be | | |
| The algorithm has not to be too strict. | | | |  | | | | | multimodal and multidisciplinary treatment is not available for many of us | | |
| The algorithm Needs to be validated with the treatment effects of the patients | | | |  | | | | | Many of the proposed steps are not accessible in this order in the NHS due to local commissioning pathways already in place, making implementation difficult. | | |
| It is difficult to truly know how other services involved with healthcare will use the algorithm, so the answers end up being an educated guess | | | |  | | | | | This is an excellent pathway if all elements of services are available in a timely fashion. There is no indication of time frames, this is relevant as there are loooong w/l for all aspects of these services. | | |
| Flexibility needs to be agreed if used to commission services - clinical expertise and concern must also be taken into consideration for individual cases. | | | |  | | | | | Patients usually appreciate knowing the rules supporting the condition pathways, but not the waits for elements to be put in to action IAPT, talking services, Pain clinics etc. able to manage the psychological programme are limited and need to be set up before this can start. | | |
| Commissioning and Policy services will need spinal specialists on their INNF panel to help guide them with their criteria based access and prior approval etc pathways. | | | |  | | | | |  | | |
| **Other themes** | | | | | | | | | | | |
| **Lumbar spinal stenosis patient profiles (phenotypes A,B,C)** | | **Additional details relating to assessment and diagnosis** | | | | | **Additional details relating to treatments and timelines** | | | **Presentation/ layout** | |
| It's the LSS LBP only patients I most struggle to manage and could benefit from treatment/management guidelines. The algorithm is only for patients with peripheral symptoms. Also often the ration of back to leg pain influences treatment decisions | | Ultimately one of the biggest issues is that I suspect that LSS is being underdiagnosed in general practice, so earlier and more frequent recognition would be imperative, along with ensuring that clinicians have easy access to treatment algorithms and clinical decision aids to facilitate shared decision making with patients. | | | | | Would like to see more detail on the non-surgical offer e.g. settings of intervention (primary vs secondary care) for rehab and expected timeframes | | | Too much info in too small a space | |
| I also find the clear phenotypes very helpful. | | I wonder if vascular claudication needs a mention on the pathway given the difficulty differentiating from neurogenic claudication. We get a number of referrals from vascular consultants and likewise we refer to them or request vascular screening. | | | | | Could do with a (flexible) timeline to be more useful (accepting variability). | | | Perhaps too many 'may' and 'consider' but I get why you have them - if you can be more definite, be so. | |
| For me it's difficult to approach the A, B, and C types by means of this standardised algorithm. | | Early diagnosis and intervention is most helpful clinically. | | | | | No timeframe given. | | | It is confusing, too detailed, and not applicable | |
| There is confusion in the Type C terminology between radicular pain (chemically-mediated sensitisation of a spinal nerve root) and radiculopathy (mechanical compression of a nerve root). Radicular pain is not due to nerve root compromise as indicated in the text. The interventions associated with each of these are different and therefore the conditions require differentiation. | | The goal should be early diagnosis… | | | | | Should consider including timelines for stepping; i.e., how long does one try Step 1 before progressing to Step 2, etc.? | | | Point system would be better, unclear algorithm. | |
| Type A-C have a lot of crossover and may be present simultaneously | | I believe that there needs to be an additional step, before self-management, where the person is assessed by a primary healthcare provider, before self-management. | | | | | There are no timeline (which is hard to predict), and therefore, becomes more of an academic exercise to show what the pathway could look like. | | | Incorrect use of capital letters irritating. Colour scheme is not assertive-doesn’t draw your eye along the pathway. | |
| Where is the way for type b described? | | It may be helpful to have a table that compares and contrasts to vascular claudication so that general practice MDs can remember how they can seem similar but have key clinical differences. | | | | | How long does a clinician / patient trial these elements before review and staging for stepped pathway. | | | Nobody in the clinical setting has time to read and understand such a complicated algorithm. | |
| Does not differentiate between lateral and central. | |  | | | | |  | | | too complex... make it simpler | |
| Three types of stenosis symptoms did not need classified for this algorithm. They are not used to direct care. No indication of first vs recurrent episodes, vs constant intrusive symptoms. First episodes have a good natural history for spontaneous resolution (see sport results). | |  | | | | |  | | | Well laid out and easy to follow | |
| More detail about type b sensory group would be useful. Would it useful to specify reduction in walking distance due to leg pain? | |  | | | | |  | | | algorithm to complicated | |
| The drawback will be that not all patients fit snuggly into one of the categories | |  | | | | |  | | | I'm not sure how the arrows/direction of travel pointing to "If persistent or recurrent symptoms" differs from the arrows that point to the next step from the main dialogue box | |
| When does neuro deficit become more important factor, | |  | | | | |  | | | The matrix seems cumbersome | |
|  | |  | | | | |  | | | Looks good. Make it 3rd grade level reading for the public to understand | |
|  | |  | | | | |  | | | I found the algorithm a bit complex to read. a simplified version would be better | |
|  | |  | | | | |  | | | The flow diagram is difficult to follow, particularly how they appear to be able to step on and off at any level. | |
|  | |  | | | | |  | | | I think it is hard to form an algorithm for such a varied and complex condition. I think it is a good attempt but that some practitioners may be deterred by relatively large amount of information | |

*Notes: Typographical errors in participant responses have been corrected; Categories for comments are derived from inductive thematic analysis.*

**Supplementary Table 8: Themes from respondents’ comments on reasons why algorithm not useful each stakeholder group**

| **Private** | **Specialist spinal /secondary care healthcare clinicians** | **Primary care/GP** | **Commissioners** | **Policy makers** | **Insurers** | **Researchers** | **Shared decision aid for patients** |
| --- | --- | --- | --- | --- | --- | --- | --- |
| probably not useful as likely to have their own preferred algorithm | probably not useful as these stakeholders are familiar with condition, and would base treatment decisions on clinical and imaging findings | not useful for most, as 'I don't like algorithms' | not useful for most - commissioners use algorithm to control healthcare clinicians | probably not useful - only useful for people dealing with patients | probably not - only useful for people dealing with patients | not useful for most, as 'I don't like algorithms' | completely useless - healthcare clinicians propose/decide pathways (with patient) |
| not useful for most as nothing new for these stakeholders | probably not useful as CPPP may not be acceptable to patients in Step 2 | not useful for most as treatment decisions need to be made individually | not useful for most, as 'I don't like algorithms' | not useful for most, as 'I don't like algorithms' | completely useless - insurers would not use algorithm to control/ advise healthcare clinicians | Completely useless - what would the research be? | not useful for most, as 'I don't like algorithms' |
| Completely useless as self -advice and psychological multimodal rehab is not possible in Germany for ambulant patients. | probably not useful as likely to have own preferred algorithm | probably not useful - not necessary | probably not useful - need further insight to be able to tailor their offers | probably not useful - need further insight to be able to tailor their offers | not useful for most, as 'I don't like algorithms' | not useful for most - too conservative | not useful for most - too conservative |
| not useful for most, as 'I don't like algorithms' | probably not useful - as likely following a similar pathway already | probably not useful - very busy / complex flowchart/not user friendly | completely useless - too complicated | completely useless - gives impression that surgery only indicated in rare cases after numerous conservative interventions | not useful for most - should not be involved in decision process | *unsure as they go round in circles | *unsure - depends on specific simplified version |
| not useful for most as looks too complicated | completely useless as nothing new for these stakeholders (may work for GPs/physios) | completely useless - too complicated | probably not useful - no funding available for this condition | probably not useful - no funding available for this condition | Probably not useful. Could be used as a tool for reimbursement/ negotiation | *Unsure if useful as depends on familiarity with condition and treatment results | *not useful as current algorithm is not complicated (therefore don’t need a simplified version) |
| not useful for most as treatment decisions need to be made individually | probably not useful - patients have already been through incomplete/ unstandardised conservative care when arrive in specialist clinic. Insufficient time for advice on self-management, and no links to services providing steps 1/2 | not useful for most - too conservative | not useful for most - too conservative | not useful for most - too conservative | completely useless - will lead to conclusion that surgery not necessary at all | *probably useful - to contribute to evidence | *probably not useful - as already simple |
| probably not useful - not necessary | not useful for most as simply describes what spinal specialists should already know. Also psycho-behavioural approach only appropriate in very specific cases | probably not useful - self management will not help if there is a structural problem | probably not useful - concerns as diagnosis and treatment are complex | not useful for most - would probably limit patient access to care | not useful for most - too conservative |  | *useful for most. Well trained physicians have skills to diagnose type and severity of stenosis |
| probably not useful - very busy / complex flowchart/not user friendly | not useful for most, as 'I don't like algorithms' | probably not useful as patients will get bogged down in useless conservative pseudo-treatment | not useful for most - most people in my country of practice (Africa) would not understand or use | completely useless - policy-makers out of touch with reality | not useful for most - would probably limit patient access to care |  | *extremely useful as simple, and patients and some clinicians often only think of surgery for LSS. This demonstrates other options |
| completely useless - too complicated | not useful for most as looks too complicated | *unsure if useful as it may be misleading and denying patients access to valid treatment options | not useful for most - would probably limit patient access to care | completely useless as they will look at cost not quality | completely useless as they will try and stop appropriate treatment |  | *probably useful but is no more than a written outline of a PARQ discussion (procedures, alternatives, risks, questions) and there are other resources that already do this |
| not useful for most - too conservative | not useful for most as treatment decisions need to be made individually | *probably useful as awareness of algorithm may help referral pathways | completely useless as they will look at cost not quality | *unsure as above (i) risk of becoming a tick-box exercise, ii) steps not appropriate for all patients iii) difficult to capture multifactorial complexity of treatment decisions in an algorithm | *extremely useful - possibly to restrict reimbursements |  | *extremely useful if using very simple terms |
| probably not useful - self management will not help if there is a structural problem | probably not useful - not necessary | *extremely useful, depending on level of experience | probably not useful - i) risk of becoming a tick-box exercise, ii) steps not appropriate for all patients iii) difficult to capture multifactorial complexity of treatment decisions in an algorithm | *useful for most - but need to ensure flexibility for individualised care | *probably useful, but have to be careful that they will apply it too strictly |  | *extremely useful, simple algorithm but too complex to follow easily |
| completely useless as patients present with severe symptoms | not useful for most - as too complicated, and patients in this setting are in later stages needing decision for/against surgery | *probably useful, but a limitation is lack of access to ESI in some locations | *useful, because clear understanding of management and resources needed | *probably useful as awareness of algorithm may help referral pathways |  |  | *extremely useful if similar to versus Arthritis SDM tools |
|  | not useful for most - algorithm very unspecific |  | *probably useful as awareness of algorithm may help referral pathways | *probably useful, but have to be careful that they will apply it too strictly |  |  | *extremely useful, would add timings |
|  | not useful for most - too conservative |  |  |  |  |  |  |
|  | probably not useful - self management will not help if there is a structural problem |  |  |  |  |  |  |
|  | completely useless as patients present with very severe symptoms |  |  |  |  |  |  |
|  | Specialist: not useful for most as should already be using an equivalent algorithm |  |  |  |  |  |  |
|  | not useful for most as these stakeholders should be familiar with LSS management and treatment options (and therefore not need algorithm) |  |  |  |  |  |  |
|  | *unsure - helpful for trainees but otherwise it is a common algorithm |  |  |  |  |  |  |
|  | *unsure if useful - as surgery should be informed choice for patients at every point of the pathway |  |  |  |  |  |  |
|  | *extremely useful due to clarity and use of standardised phenotypes, and staged approach |  |  |  |  |  |  |
|  | *probably useful/useful for some - but you would expect exert/specialist clinicians to have the expertise/knowledge without consulting algorithm |  |  |  |  |  |  |

*Note: Tyographical errors in participants’ responses have been corrected; *additional comments from participants included for completeness – the survey requested explanations only from participants who had responded that the algorithm was not useful, but some participants rating the algorithm as useful or unsure chose to add these comments.*


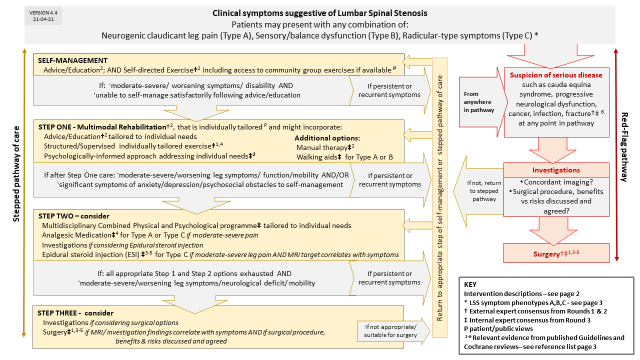

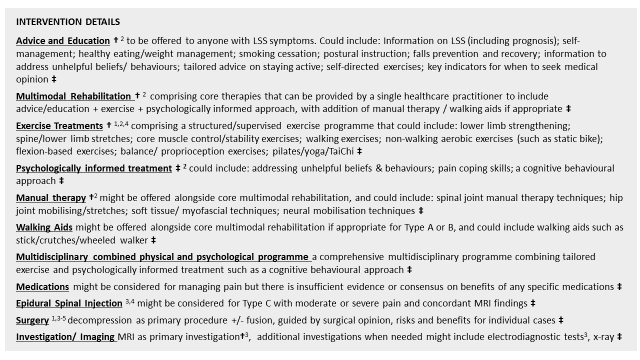

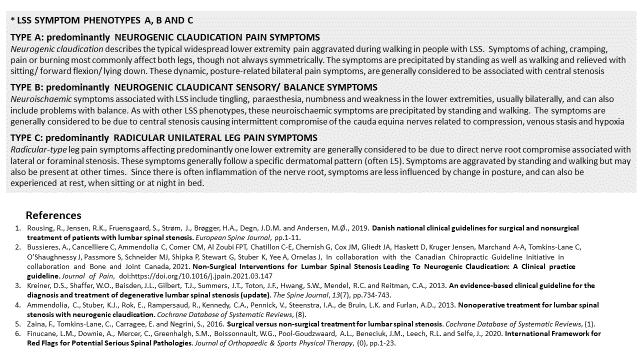
 **Supplementary Figure 1: Draft algorithm shared in Round 4**
